# Supplementary material for: Knowledge + Innovation = Power: A protocol for implementing Aboriginal data sovereignty in an Aboriginal Medical Service for service delivery reform
Source: PLoS One. 2025 Oct 13;20(10):e0328588. doi: 10.1371/journal.pone.0328588 (PMC12517504; doi:10.1371/journal.pone.0328588)
Supplement: S1 Appendix — (PDF) [file pone.0328588.s001.pdf]

Appendix 1. Preliminary survey and Interview questions
(Final questions to be determined by the Community Panel co-researchers and SWAMS Research team)

Survey questions

- Likert scale questions
  - To what extent has your knowledge of research increased?
  - To what extent confidence in discussing research increased?
  - To what extent has your knowledge of data collection, analysis, reporting and advocacy increased?
  - To what extent has your understanding of local HWB priorities/needs increased?
  - To what extent has your understanding of local HWB aspirations/hopes increased?
  - To what extent has your knowledge of population health data and data custodians increased?
  - Did you feel that the population health data resources provided were easy to understand?
  - Did you feel that the data visualisation resources provided were useful?
- Open-ended responses – free text spaces below each of above questions plus an additional space for "Other information you would like to tell us"

Interview and yarning circle questions
<u>1. Community Research Panel</u>

Preliminary yarn regarding extent of involvement.

- Can you tell me/us about your experiences during the formative phase of the CRP?
- Can you tell me/us about your experiences during the process of setting up the evaluation research (determining research questions, how to conduct surveys etc)?
- Where there any challenges? Can you tell me/us more about those?
- What have been the most important outcomes/learnings for you? Can you tell me/us more about those?
- To what extent do you feel our work has had impact on SWAMS policy and practices? Or has the potential to? Can you tell me/us more about that?

<u>2. SWAMS staff, including managers and board members</u>

Preliminary yarn regarding extent of involvement.

- Can you tell me/us about your perceptions regarding this project, including the formation of the CRP and its implementation of research?
- What do you see as the benefits/opportunities from having a CRP and its outcomes?
- Where there any challenges? Can you tell me/us more about those?
- What have been the most important outcomes/learnings for you regarding the CRP and its outcomes? Can you tell me/us more about those?
- To what extent do you feel the work of the CRP has had impact on SWAMS policy and practices? Or has the potential to? Can you tell me/us more about that?
- What do you see as the enablers and barriers to implementing CRP decisions and priorities including systemic issues such as data tools and existing measures?
